# Nanotube Alignment and Surface Chemistry in Altering Water and Salt Permeabilities for Imogolite-Polyamide Membranes

**DOI:** 10.3390/membranes16010020

**Published:** 2026-01-01

**Authors:** Savannah Bachmann, Jonathan Brant

**Affiliations:** Department of Civil and Architectural Engineering and Construction Management, University of Wyoming, 1000 E University Avenue, Laramie, WY 82071, USA; sbachma1@uwyo.edu

**Keywords:** imogolite nanotubes, nanocomposite membrane, desalination, water permeance, salt permeability

## Abstract

Reducing the specific energy consumption of reverse osmosis (RO) processes motivates the development of new membrane materials that have enhanced water permeability while maintaining low salt permeability (high rejection). Nanocomposite membranes have shown great promise in achieving these goals, particularly those using nanotubes as fillers. Here, we report on the relationships between the orientations and surface functionalities of imogolite nanotubes (INTs) with water and salt permeabilities for polyamide nanocomposite membranes. An external electric field was used to manipulate the INT orientation within the polyamide active layer. The INT interior and exterior chemistries, respectively, were made hydrophobic using methyl triethoxysilane as a precursor during INT synthesis and post-synthesis modification with alkali-phosphate groups. Irrespective of nanotube orientation or surface chemistry, membrane permeance increased from 0.3 to ≥1.0 L m^−2^ h^−1^ bar^−1^. A salt permeability comparable to the conventional polyamide membrane was maintained by making the INT pore throat hydrophobic. These findings indicated that salt rejection could be tailored by manipulating the INT interior surface chemistry without sacrificing water permeability.

## 1. Introduction

Cadotte patented the first thin-film reverse osmosis (RO) membrane in 1980 [1]. Since then, RO has been used worldwide to meet growing freshwater demands and now accounts for the largest share of the desalination technology market. Although many improvements have been made in membrane design, the need to lower the specific energy consumption (SEC) of RO processes remains [2,3]. Membrane permeability accounts for about 25% to 50% of the SEC in seawater (SWRO) and brackish water (BWRO) systems. Many recent studies have focused on increasing the water permeability of RO membranes while maintaining low salt permeability, leading to several significant advances.

Nanocomposite membranes have been studied to improve water permeance and reduce fouling. Nanotubes, whether carbon-based or other types, are a common nanofiller that has received extensive attention. Imogolite nanotubes (INTs) are a specific type of nanotube that have been examined [4,5]. INTs are known for their high aspect ratios, rigidity, and unique polarities, which allow their orientation to be controlled using electric fields [6]. Silanol groups are found on the interior pore walls of INTs, while aluminol groups are on the exterior surface [7]. These chemistries render the INTs hydrophilic and charged, aiding water diffusion and solute rejection via Donnan exclusion. Additionally, the physical and surface chemical properties of INTs can be modified. For instance, the pore throat diameter can be increased by replacing silica with germanium as a precursor during synthesis [8], which also extends the length of the INTs from approximately 100 nm to 1 µm [8]. Such structural changes may enable tuning of solute rejection through steric interactions. The surface chemistry of INTs can also be made hydrophobic by substituting tetraethyl orthosilicate (TEOS) for methyltriethoxysilane (TEMS), which also increases the pore throat diameter from 1.0 to 1.7 nm [9,10]. The ability to alter the physical and surface chemical characteristics of INTs offers a valuable opportunity to study how these factors influence water diffusion in nanocomposite membranes.

Water transport through nanotubes is expected to be enhanced by a constrained movement that eliminates random diffusion through a dense thin film [11,12,13,14] and frictionless transport through hydrophobic nano-confinements, such as nanotubes. Constrained movement refers to restricted molecular motion that forms linear and strongly bonded water or “water wires.” Water wires generate an excess proton that migrates along the wire of bonded water molecules [15]. Essentially, individual water molecules do not move as a unit; instead, hydrogen (proton) translocation diffuses through the central oxygen, driving water molecules through the nanotube. This proton migration across water molecules is known as the Grotthuss mechanism and has been modeled in carbon nanotubes [16] and INTs [13]. Contrary to hydrophobic nanotubes and diffusion theory via proton exchange, hydrophilic surfaces exhibit strong adhesion to water molecules. Barona found that INTs increased the water permeability of a polyamide membrane [14]. Similarly, Shi et al. studied INT nanocomposite membranes for forward osmosis applications [17]. They found that water diffused spontaneously and preferentially into the INTs, resulting in a flux of 28.4 Imogolite nanotubes; desalination; water permeance; salt permeability without applied pressure. Ion selectivity was also modeled; chloride ions were rejected due to coulombic interactions, while sodium ions were electrostatically bound to the INT’s walls, where they remained without diffusing through the membrane until saturation occurred. In a later study, Li and Brant found that incorporating INTs into polyamide thin films increased membrane permeance by adjusting the polymer’s cross-linking density and expanding the free volume within the membrane [5]. Using NMR relaxometry, Belorizky determined that the one-dimensional diffusion rate of water through INTs was 0.5 × 10^−2^ cm^2^ s^−1^, which was four times slower than that for free water [18]; however, Creton distinguished between water molecules inside the INT and those diffusing along its outer surface. The slower rates occurred inside the tubes, and the faster rates occurred across the outside perimeter [13]. These diffusion rates were attributed to aluminol (Al-OH) groups on the outer surface, which impart a positive surface charge. In contrast, the internal surface is covered with silanol (Si-OH) groups, which exhibit a slightly negative charge [19]. The electrostatic interactions at these two interfaces dictated the water diffusion rate and the extent of ion selectivity for sodium and chloride.

Hydrophilic surfaces attract and adsorb water molecules that diffuse through the nanotube in response to chemical gradients via Fickian diffusion. There are two main differences between water diffusion in hydrophilic and hydrophobic nanotubes: first, the interaction between water and the nanotube’s walls; second, the energy produced by water molecules inside the nanotube’s pore throat. A hydrophobic surface repels water molecules, forcing the water into a linear chain that is optimal for proton shuttling. In contrast, hydrophilic INTs can absorb up to a quarter of their mass in water. This adsorption slows down molecular diffusion. The entropy generated from enthalpic penalties in confined spaces might not exist in hydrophilic nanotubes, as water can dissociate and freely adsorb onto the INT’s walls along with other water molecules. This lower-entropy state removes the cascading energy system observed in hydrophobic nanocavities. Without this additional energy source, molecular diffusion depends solely on chemical gradients. Despite significant progress in this field, questions remain about how, or if, the internal and external surface chemistry of the INTs affects water permeance and salt rejection in nanocomposite membranes. An additional factor to consider for nanofillers and their effect on membrane performance is their impact on the structure and cross-linking density of the salt-rejecting layer. Xue et al. found that whether 2D MXene nanosheets were dispersed in the organic or aqueous phase affected the thickness and cross-linking density of the polyamide structure [20]. Dispersing the nanosheets in the aqueous phase resulted in a thinner polyamide layer with nano-voids, leading to higher permeance and lower salt rejection. Conversely, dispersing the nanosheets in the organic phase led to higher cross-linking densities, which in turn caused lower permeance and higher salt rejection values. These studies highlight the many ways nanofillers can influence membrane performance.

This work investigated how altering the hydrophobicity of the pore throats in INTs influences water and salt permeabilities. INTs were embedded into polyamide thin films formed on polysulfone supports. It also controlled the orientation of the INTs within the polyamide thin film using an alternating electrical current. The addition of INTs, regardless of their orientation or surface chemistry, enhanced water permeance. Sodium permeability stayed the same for INTs with hydrophobic pore throats compared to the pure polyamide membrane, even though the pore throat diameter increased relative to their hydrophilic versions.

## 2. Materials and Methods

### 2.1. Chemicals and Reagents

Test solutions were prepared with ultrapure water (Milli-Q^®^ IQ, Millipore Sigma, Burlington, NJ, USA) having a resistivity of 18 mΩ∙cm and an unbuffered pH of 4.5–5.0. INTs were synthesized using aluminum hexahydrate, AlCl_3_·6H_2_O (Acros, 99%) and tetraethyl orthosilicate (TEOS), SiC_8_H_2_O_4_ (Millipore Sigma, Burlington, NJ, USA, purity = 98%), or methyl triethoxysilane (TEMS) CH_3_Si(OC_2_H_5_)_3_ (Millipore Sigma, 99%). Basification was performed using sodium hydroxide (Millipore Sigma, 1.0 N); acidification was carried out with either hydrochloric acid (Millipore Sigma, 1.0 M) or acetic acid (Millipore Sigma, ACS Reagent Grade purity ≥ 99.7%). INTs were dialyzed using Spectrum™ Labs Spectra/Por™ 6 dialysis membranes having a molecular weight cutoff of 1000 Daltons. Post INT phosphorylation used ammonia (Millipore Sigma, anhydrous purity ≥ 99.98%), mono-n-dodecyl phosphate (Alfa Aesar™, Ward Hill, MA, USA, technical grade = 90%), isopropanol (Alfa Aesar, Ward Hill, MA, USA, ACS 99.5%), sodium acetate buffer, pH 5, and tetrahydrofuran (Millipore Sigma, anhydrous 99.9%). The support membrane was polymerized using a mixture of polysulfone (PSU, Millipore Sigma, Mn ~22,000 by MO, beads) and N-methyl-2-pyrrolidinone (NMP, Millipore Sigma, anhydrous, 99.5%). Polyamide thin films were formed using m-phenylenediamine, MPD (Millipore Sigma, flakes 99%), 1,3,5-benzenetricarbonyl trichloride, TMC (Millipore Sigma, 98%), and n-Hexane (Millipore Sigma, 99%).

### 2.2. Synthesis of Imogolite Nanotube (INTs)

The Farmer’s method was used to synthesize the INTs [21]. A 1.5 mM aqueous solution of TEOS, was added dropwise into a 3.0 mM ultrapure water solution of AlCl_3_·H_2_O at a rate of 2.5 mL min^−1^ under continuous mixing to hydrolyze the aluminum salt. This yielded a 2:1 molar ratio of Al to Si, with Si concentration ≤ 2 mM. This avoided precipitating insoluble silica, which cannot bond to aluminum gibbsite sheets [22,23]. The 2:1 Al/Si solution was mixed at room temperature (~21 °C) for 45 min, allowing the hydrolysis reactions to reach equilibrium before raising the pH [22,24]. Next, basification was induced dropwise with 0.1 M sodium hydroxide solution until a [OH]/[Al] ratio of 2:1 was achieved, corresponding to a pH of 5.0. The mixture was acidified to pH 4.5 using a 2:1 mixture of 0.2 M CH_3_COOH and 0.1 M hydrochloric acid. The resulting solution yielded proto-imogolite platelets. The self-assembly into tubes and elongating growth of INTs was initiated by heating the mixture to 95 ± 2 °C for 5 days. Under these conditions, the suspended platelets self-assemble into gibbsite sheets consisting of Al-OH that bond with silanol, Si-OH, across the inside of the sheet, causing the gibbsite sheets to curl into tubes due to the differing bond lengths O-Si-O, <3 Å and 3.2 Å for Al-O-Al [25]. Finally, the mixture was purified using a 1 kDa cutoff dialysis membrane and ultrapure water, which was replaced regularly. Dialysis continued until the conductivity of the ultrapure water was ≤5 µS/cm. The purified mixture was stored at 4 °C until lyophilization. A Labconco™ FreeZone Plus (Labconco Corporation, Kansas City, NJ, USA), 2.5 L lyophilizer was used to freeze-dry the INTs into a cotton-like substance. The aqueous-INT solution was poured into a clean Labconco™ fast-freeze flask and frozen overnight. The lyophilizer was set to 0.5 mbar and −48 °C. Once the appropriate temperature and pressure were reached, the frozen flask containing the INT mixture was connected to the lyophilizer until all ice was removed, leaving only powdered INTs. The powdered INTs were collected in a sterilized MTC Bio 50 mL polypropylene test tube and stored in a desiccator cabinet.

### 2.3. Surface Modification of INTs

INT surface modifications involved substituting precursors during synthesis for inner-diameter changes and performing post-synthesis grafting of functional groups on the outer surface. The inner INT surface was functionalized by replacing the silica precursor, TEOS, with TEMS, which contains methyl groups that replace the three hydroxyl groups on the inner silanol structure [26]. The subsequent steps followed the same procedure to produce unmodified INTs. It should be noted that unmodified INTs are called INTs, while TEM-substituted INTs are named Me-INTs. The outer surfaces of the INTs were modified through phosphorylation using precipitated ammonium salts containing hydrophobic, alkyl-phosphate groups. Grafting alkyl-phosphate groups from dodecyl phosphate powder cannot be achieved by simple substitution or addition because this powder is water-insoluble. Instead, these groups were synthesized following the protocol developed by Hofer [26]. In this process, 2 g of water-insoluble dodecyl phosphate was dissolved in 200 mL of 2-propanol and heated in a jacketed reactor at 82 °C for 6 h. Afterward, the solution was removed from the heat, and 6 mL of ammonium was added. The hot solution was then cooled in an ice bath, causing the immediate precipitation of the water-soluble ammonium salt [DDPO_4_(NH_4_)_2_], which generally binds to transition metals [23,26]. These salts were filtered through a 0.45 µm glass fiber filter and rinsed five times with chilled 2-propanol. The collected salts were heat-cured at 60 °C under a vacuum of 10 mbar, yielding approximately 1.5 g of dried ammonium salts [DDPO_4_(NH_4_)_2_].

DDPO_4_(NH_4_)_2_ was grafted to both imogolite groups, TEOS-INTs and Me-INTs, according to the procedure outlined in Ma et al. [27]. 300 mg of freeze-dried TEOS-INTs and Me-INTs were dissolved in separate volumes of pH 5.0 buffered 100 mL ultrapure water, bath sonicated for 1 h, and left to mix on a rotary shaker for 48 h, allowing for the preferential chemisorption of dodecylphosphate [DDPO_4_(NH_4_)_2_] onto the aluminol groups. During this process, the phosphate forms a monolayer of strongly bonded metal (aluminum)-oxygen-phosphate from hetero condensation and coordination across the exterior of the INTs. Phosphate groups are not susceptible to condensation reactions when attached to silanol groups. Thus, the silanol phosphorylation does not persist during the chemical process, leaving only the outer surface of the INTs covered with phosphate groups. Once equilibrium has been reached, the phosphate-grafted INT-groups are rinsed with 200 mL of a 1:1 (*v*/*v*) solution of ultrapure water and tetrahydrofuran through a ceramic membrane (Sterlitech Corporation) with a molecular weight cutoff of 150 kDa. The retained powder was collected and lyophilized at −48 °C and 0.5 mbar for 24 h before use. The phosphate-grafted nanotubes (P-TEOS-INT and P-Me-INT) were dissolved in n-hexane at a concentration of 0.1% *w*/*v* and sonicated for 30 min immediately before forming the nanocomposite membranes (TFN-A-Me, TFN-UA-Me, and TFN-A-TEOS).

### 2.4. Physical and Surface Chemical Characterization of INTs

The zeta potentials (electrophoretic mobilities) of the unmodified and modified INTs in relevant solvents were characterized using a ZetaSizer Nano ZS dynamic light scattering system (Malvern Panalytical). All measurements were performed using a background electrolyte solution containing 1 mM potassium chloride (T = 25 °C). The electrolyte solution was filtered through a 0.22 µm syringe filter before dispersing the sample INTs. Measurements were taken across a pH range of 3 to 11, adjusted with hydrochloric acid and potassium hydroxide. Zeta potential was calculated from the measured electrophoretic mobility using the Smoluchowski equation [28,29]. The reported zeta potential values are averages across 15 samples.

The lengths of the modified and unmodified INTs were measured using transmission electron microscopy analysis (TEM, Hitachi H-7000, Hitachi, Hillsboro, MO, USA). TEM samples were prepared by placing a droplet of the relevant INT-aqueous solution onto a copper grid disk (CF-1.2/1.3-4 Cu-50, Electron Microscopy Sciences, Hatfield, PA, USA). After drying, the grid was inserted into the TEM and imaged using a beam voltage of 75 kV and a current of 25 µA. Images were analyzed with Image-J V1 software (National Institutes of Health, USA). Six unique samples containing at least 50 INTs each were used to determine the average lengths.

The chemical functionalities of the unmodified and modified INT surfaces were characterized using Fourier Transform infrared microscopy (FTIR, Nicolet iS50 FTIR Spectrometer, Thermo Fisher Scientific, Waltham, MA, USA). FTIR analyses were performed on lyophilized, compacted INT disks to identify the key signatures of natural imogolite and the grafted functional groups, including alkyl-phosphates and methyl groups. Three unique samples of each INT type were analyzed, with 42 scans per sample. The background and water peaks between 2500 and 2000 cm^−1^ were removed to eliminate interference.

### 2.5. Membrane Synthesis

All membranes comprised a polyamide active layer and a PSU support. A summary of the membrane types evaluated in this study is given in Table 1. The PSU solution consisted of a 15/85 ratio of PSU beads (22,000 Mn) to 1-methyl 2-pyrrolodine (NMP). The PSU-solution was mixed for 24 h in a rotary mixer and then bath sonicated for 2 h under vacuum to degas it. The PSU membrane was cast using a motorized casting system (Automatic Film Coater MSK-AFA-11, MTI Corporation, Richmond, USA) on a borosilicate glass plate. The membrane’s thickness was 350 µm. After casting, it was immediately immersed in a coagulation bath containing ultrapure water to initiate polymerization. The membrane remained in the water bath for 30 min, then rinsed with ultrapure water for 3 min. Membranes were stored in ultrapure water for at least 24 h before use. All solutions used for membrane synthesis were maintained at room temperature (22 °C).

The polyamide active layer was polymerized onto the PSU support. The wet PSU-support was cut into a 12.7 cm square, then soaked in a 2% *w*/*v* solution of MPD for 8 min to saturate it with amine groups. The MPD-soaked PSU was then taped to a glass plate using FrogTape^®^. A rubber roller was pressed firmly over the PSU membrane to remove any excess MPD solution. A 6 mL volume of 0.1% *w*/*v* solution of 1,3,5-Benzenetricarbonyl trichloride (TMC) was poured onto the PSU membrane (167.3 cm^2^) and dried for 2 min. The membrane was placed inside an oven for 10 min at 75 °C, rotating front to back every 5 min to ensure even heating. The cured membrane was cooled at room temperature for 15 min, then rinsed with ultrapure water and stored in it. The embedding of unaligned INTs followed the same protocol, except that 0.1% *w*/*v* of INTs was added to the *n*-hexane, then probe sonicated for 1 h at 20% amplitude. This amplitude was selected as it resulted in improved dispersion of the INTs in the solvent, but it was not strong enough to degrade their structural integrity, as confirmed by TEM imaging of pre- and post-sonication samples. This was followed by the addition of 0.1% *w*/*v* TMC to the solution, which was sonicated again for an additional hour at 20% amplitude. The 0.1% INT/TMC solution was sonicated for 15 min before forming the membranes to ensure INT dispersion. Membranes were kept in ultrapure water until use.

A procedure for aligning INTs in polyamide thin films was previously developed and reported on [5]. The reader is referred to this publication for a more in-depth analysis confirming INT alignment in the polyamide film. The alignment apparatus included a polytetrafluoroethylene (PTFE) block that housed a 2.54 cm-thick, electrically conductive copper plate. A 4.8 cm ledge was present to create a shallow bath around the copper block, preventing the polymerization solutions from coming into direct contact with the top copper plate. The copper plate capacitors were charged for 15 min using a 110 V banana jack electrical lead connected to an AC converter box that delivered 15 A at a frequency of 9 kHz. After charging the copper plates for 15 min, the top (negative plate) and bottom (positive plate) were separated, and the glass plate containing the MPD-soaked PSU membrane was placed on the bottom copper plate. The apparatus stayed connected to an electrical outlet during this step to reduce charge loss. Then, the membrane was polymerized while aligning INTs by carefully pouring 6 mL of a 0.1% *w*/*v* solution of both INT/TMC over the MPD-soaked PSU membrane; the top housing, which contained the negatively charged copper plate, was then lowered. The membrane was polymerized for 2 min until the solution evaporated. The two electrical connections were disconnected, the top and bottom were separated, and the glass plate holding the newly polymerized TFN was removed. The INT-aligned TFN was cured in the oven under the same conditions as the unaligned-TFN membranes, producing the TFN-A-Group membranes.

### 2.6. Membrane Surface Chemistry

The integration of the INTs into the polyamide active layer was assessed using Attenuated Total Reflectance Fourier Transform Infrared spectroscopy (ATR-FT-IR, Nicolet™ iS50, Thermo Fisher Scientific). Sample coupons were cut from randomly selected locations on membrane samples and dried for 24 h. Forty-two scans were collected at each site to build the reported IR spectra.

### 2.7. Membrane Performance Assessment

Permeance (L m^−2^ h^−1^ bar^−1^) was characterized using a dead-end filtration apparatus and ultrapure water as the feed solution. The apparatus consisted of a stirred cell (HP4750, Sterlitech Corporation, Auburn, AL, USA) with an active membrane area of 14.6 cm^2^, a compressed nitrogen cylinder supplying hydrostatic pressure, a digital pressure gauge, and a mass balance. The pressure gauge and mass balance were interfaced with a computer for real-time data collection. All membrane samples were compressed at 35 bar for 1 h before starting the permeance measurements. Permeance was calculated from a linear regression analysis of water flux as a function of feed pressure over a pressure range of 17 to 31 bar. Water flux at each pressure step was measured as the feed pressure was increased to 31 bar and then decreased to 17 bar. The feed water temperature was 20 ± 0.5 °C. Reported values are the averages of three different membrane samples.

Solute permeability for sodium was measured using a crossflow test system operated in a closed-loop setup. Membrane samples were housed in a CFO16D test cell (active area = 20.6 cm^2^, Sterlitech Corporation). A PTFE permeate carrier and a PTFE high-foulant spacer were used in all tests. The feed solution temperature was maintained at 20 ± 0.3 °C for all tests using a heat exchanger (Polystat^®^, Cole-Parmer, Vernon Hills, IL, USA). Feed and permeate pressure, pH, electrical conductivity, and temperature were all measured using computer-interfaced sensors. Permeate flow rate (water flux) was measured with a computer-interfaced mass balance (New Classic MF, Mettler Toledo, DC, USA). Back pressure on the membrane was controlled with an actuated needle valve (Hass Manufacturing Company, Poestenkill, NY, USA). The feed flow rate was measured using a manual flowmeter. Tests were initiated by compressing the membrane sample at 50 bar for 1 h using the electrolyte solution of interest (2000 mg L^−1^ sodium chloride, pH = 7). The crossflow velocity for all tests was maintained at 0.23 m s^−1^, corresponding to a Reynolds number of 17,482 (turbulent conditions). After compression, the applied pressure was reduced to 24.12 bars for 30 min before collecting the first feed and permeate samples. Feed and permeate samples were collected at 1 h intervals over a 4 h period. Each permeate sample’s mass, temperature, pH, and electrical conductivity were manually measured throughout sampling. Sodium concentrations were measured using an ion chromatograph (Dionex, ICS-2100, Thermo Fisher Scientific). Ion concentrations were used to calculate the respective ion rejection, *r*, and the intrinsic salt permeability coefficients, *B* (Equation (1)).

(1) $$B=A\left[\frac{\left(1-r\right)\left(∆P-∆\pi \right)}{r}\right]$$

where *A* is the hydraulic permeability coefficient (permeance) for the membrane; *r* is the ion rejection value for the relevant ion; ∆*P* is the hydraulic pressure gradient across the membrane; and ∆*π* is the osmotic pressure gradient across the membrane. The hydraulic and osmotic pressures of the permeate solution were both assumed to be negligible when calculating *B*.

## 3. Results

### 3.1. Characteristics of Unmodified and Modified INTs

**Figure 1:**
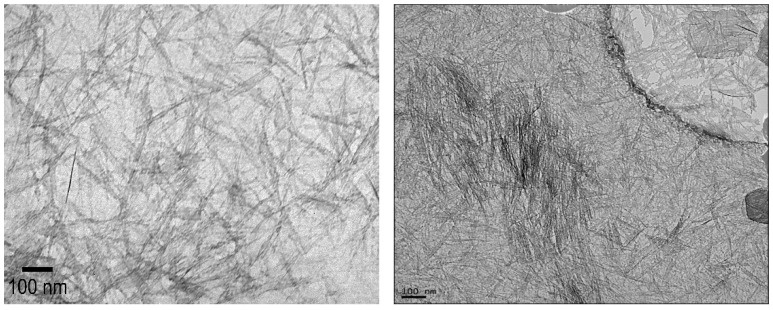
Representative HRTEM images of unmodified INTs.

Unmodified INTs had average lengths of 124 ± 35 nm (Min = 77 nm, Max = 178 nm, *n* = 15) as determined from HR-TEM images (Figure 1), consistent with previous studies using the same synthesis methodology [14,30]. This thickness is comparable to that of the polyamide active layer (~100 nm), suggesting that vertically aligned INTs could protrude from the active membrane layer. Unmodified INTs are relatively insoluble in n-hexane, which was used for synthesizing the polyamide membrane, preventing the practical synthesis INTs/polyamide composites [31]. Overcoming this limitation motivated functionalizing the outer INT surface with alkyl phosphate groups to enhance their dispersion in n-hexane and, subsequently, to form the polyamide membrane.

**Figure 2:**
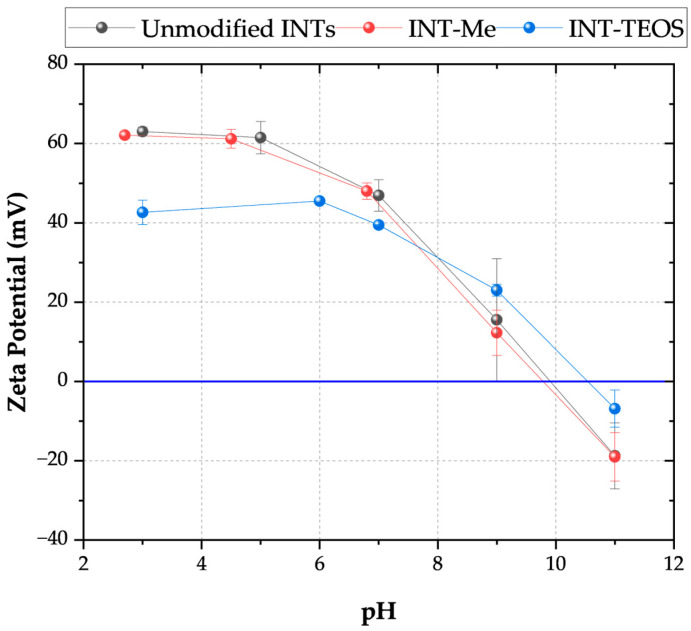
Zeta potential as a function of pH for the two types of functionalized INTs. The INT-Me had an alkyl phosphate coating on its outer wall and a methylated pore throat. The INT-TEOS INTs were functionalized with alkyl phosphate groups on their exterior and had no modifications to their pore throat. Measurements were conducted using a 1 mM KCl electrolyte solution at 20 °C.

**Figure 3:**
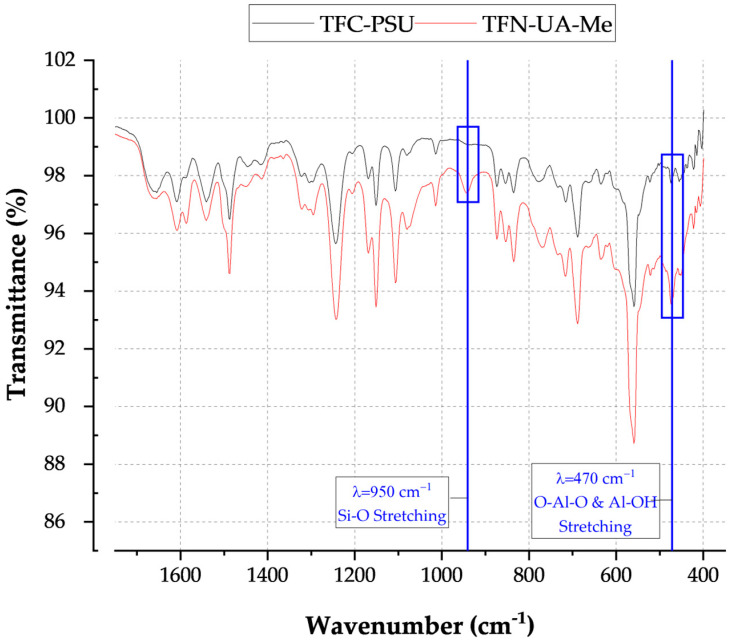
ATR-FTIR spectra for a conventionally formed polyamide membrane (TFC-PSU) and a polyamide membrane containing INT-Me nanofillers.

All INT types displayed elongated peaks from 3800 to 2800 cm^−1^, indicating the nanotubes’ aluminol exterior and silanol interior surfaces (Table 2). The phosphate groups on the TEOS-INT were identified by transmittance peaks at 1130 and 3420 cm^−1^ [14,23,31]. The peaks at 1421 and 1450 cm^−1^, respectively, confirmed the presence of methyl groups on A-Me-INT and Me-INT. The presence of the alkyl phosphate groups on the INT exterior and interior did not affect the charge character of the INTs (Figure 2). Functionalization of the exterior only (INT-TEOS) reduced the magnitude of the measured zeta potential, but the overall profile remained consistent. Confirmation of the modified INTs (TFN-UA-Me) into the polyamide structure was determined through ATR-FTIR analysis (Figure 3). Data are shown only for one type of INT nanocomposite membranes for clarity. New transmittance peaks confirmed the presence of the INTs in the polyamide at 950 and 470 cm^−1^. These peaks corresponded to Si-O and O-Al-O and Al-OH stretching, respectively. Note that both are signatures of the imogolite nanotube structures.

### 3.2. INT Properties and Membrane Performance

**Figure 4:**
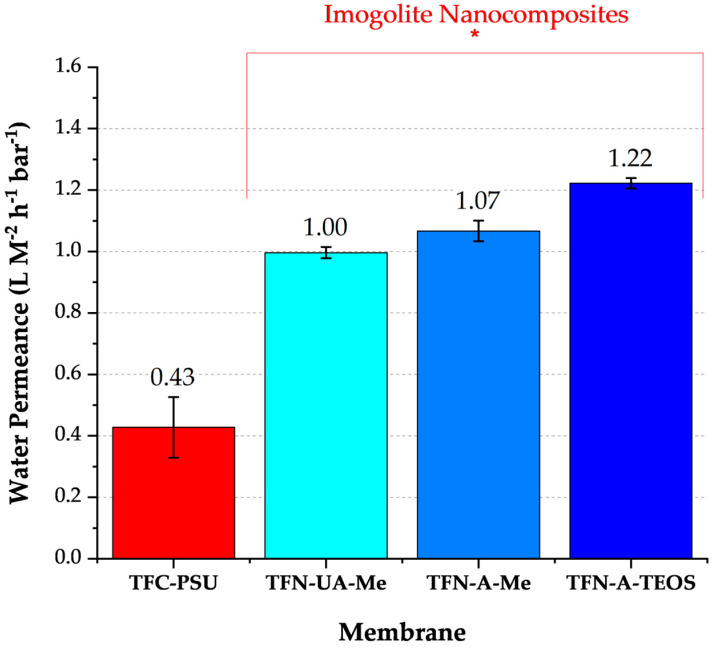
Water permeance values for a conventionally made polyamide membrane and the three INT nanocomposite membranes (feed = ultrapure water, T = 20 °C, pH = 7.0, n = 5). The asterisk bracket highlights those membranes comprised of different INTs.

The permeance values for all three nanocomposite membranes exceeded those of the conventional polyamide membrane, and the orientation and/or surface chemistry of the INTs influenced the measured water permeability (Figure 4). Various factors may affect the water permeance of nanocomposite structures. The structure of the INTs’ pore throats may act as controlled conduits, restricting the “random walk” movement of water diffusing through the polyamide [14]. Notably, each INT was characterized by pore throat diameters ≥ 1.0 nm (Table 3). In comparison, the free volume in polyamide typically ranges from 0.3 to 0.6 nm. Therefore, water molecules traveling through the INT pore throats were less confined than in the surrounding polyamide structure. Transport through the INTs may also result in a shorter travel distance to the PSU microporous support membrane. This would be particularly relevant for the INTs that were vertically oriented in the membrane (TFN-A-Me and TFN-A-TEOS). Recall that the average length of the INTs was 124 nm, and the average polyamide thickness is 100 nm, characterized by a ridge and valley-like structure. Unfortunately, because the INT outside diameter is approximately 2 nm they could not be imaged through TEM analysis. Of membrane cross-sections. Although aligning the INTs improved the water permeance, the magnitude of the improvement was not substantial. This may be attributed to a combination of factors, including a mix of aligned and unaligned INTs, which both contribute to changes in permeance.

Another factor that may contribute to the increase in permeance is changes in polymer cross-linking density surrounding the INTs [20,32]. During polycondensation between the pre-polymer groups, amine and acyl chloride, cross-linked fibers form dense, continuous structures [33]. Adding INTs to the polyamide may have altered the membrane’s cross-linking density, where the magnitude of the changes would be a function of the INT’s physical and surface-chemical properties. Earlier studies have shown that nano-fillers form regions with lower densities than the surrounding polyamide, which then serve as preferential pathways for water flow [18,19]. The way and extent to which the nanofiller affects cross-linking density depend on its surface chemistry and on its interactions with TMC and MPD. For example, hydrophilic surfaces tend to adsorb MPD, thereby decreasing the membrane’s cross-linking density and resulting in higher permeance and lower salt rejection. Conversely, hydrophobic surfaces, like the three INTs used here, tend to lower cross-linking density by hindering MPD diffusion and creating steric effects.

Conventional RO membranes are characterized by $A>L\;m^{-2}h^{-1}{bar}^{-1}$ 1 correlating with *B*-values approaching zero, or negligible salt permeability or diffusion [32]. A summary of permeability values for the synthesized membranes is provided in Table 3. Each of the nanocomposite membranes was characterized by $A>L\;m^{-2}h^{-1}{bar}^{-1}$ indicative of higher water permeabilities relative to conventionally made polyamide membranes. The TFN-A-Me and TFN-UA-Me membranes presented similarly low salt permeabilities, resulting in low B/A values. These values clearly show that water permeance remained high without compromising sodium rejection. Therefore, the observed increases in water permeance were not due to the formation of physical defects in the polyamide structure. Instead, these improvements are mainly due to enhanced molecular diffusion through the INTs.

## 4. Discussion

The *B*/*A* values for the TFN-A-Me (3.91 bar) were less than half that of the TFN-A-TEOS (9.19 bar) membrane (Table 3). The INT with the hydrophilic pore throat (TFN-A-TEOS) was characterized by a *B* (sodium permeability) approximately twice that of the INTs with the hydrophobic pore throats. Guimaraes et al. have modeled the charge distribution on the TEOS-INT and determined that the outer Al-OH bonds’ charges are Q ≈ +0.6e and −0.45e, respectively [10]. Those for the inner Si-OH Q ≈ are Q ≈ +0.8e and −0.54e, respectively [10]. Unique to INTs, these external and internal surface charges do not cancel each other out. Instead, each surface retains a net positive (Al-OH) or net negative (Si-OH) charge [10,19]. The external Al-OH groups are amphoteric and can protonate or deprotonate depending on pH [33].

At the pH of ultrapure water, pH ~ 5, the INT-Me had a zeta potential ≥ |60| mV, and about |40| mV for the INT-TEOS. The net positive charge resulted from the curvature of the aluminol, gibbsite sheets, exposing the positive valence of aluminum [19]. The gibbsite sheet curvature increased by 0.7 nm when the methyl-groups were grafted to the INT’s interior (pore throat), potentially exposing additional positively charged Al-O groups. Furthermore, the net neutral methyl groups replaced the negative hydroxyl groups across the pore throat. By increasing the pore throat diameter and replacing negatively charged sites with neutrally charged methyl groups, the overall positive charge grew. This difference in charge is suggested as one possible reason for the higher sodium rejection observed for the Me-INTs. Changes in the cross-linking densities in the polyamide films may also have contributed to the observed differences in sodium permeabilities, as observed by Hue et al. [20]. Increases in INT diameter and contributions from interactions between the different functional groups of the INTs may have favored minor changes in cross-linking density, resulting in the formation of nano-voids that facilitate greater sodium passage, as was observed for the INT-TEOS; however, the water permeance for these membranes was comparable to the other nanocomposites indicating that any changes in cross-linking density were not sufficient enough to produce substantial preferential water flow paths.

Size exclusion should also be considered when explaining the differences in sodium rejection between the two types of INTs. Notably, grafting methyl groups onto the INT pore throat increased the Me-INT internal diameter from 1.0 nm to 1.7 nm [9]. The sodium ion has a hydrated diameter of 0.358 nm, allowing it to enter the pore throats of the various INTs tested here without incurring the energy penalty associated with dehydration. It is worth noting that this penalty may have resulted from sodium entering the polyamide structure. The main difference between these INT-types is their surfaces: TEOS-INT shows an affinity for absorbing sodium’s hydration shell, which reduces the effective radii to the crystalline, Stokes’ radii of 0.184 nm [34]. Ionic interactions are crucial for determining diffusion tendencies and rates and are examined here using the sodium ion. The decrease in the size of the sodium ions and the attraction between hydrated sodium ions and the hydrophilic surface work together, leading to higher salt permeability. Additionally, the exposed hydroxyl groups present across the TEOS-INT increase water and salt sorption compared to the methyl groups, which are classified as an acetyl organic group [35]. If there is no defect in the TFN-A-TEOS, the salt passage could be explained due to the INT’s affinity for water, resulting in increased water and salt permeabilities. Further support for this theory comes from the observed *B*/*A* value for the TFN-UA-Me containing the same Me-INT. The relatively lower value is associated with reduced salt permeability and increased sodium rejection compared to the TFN-A-TEOS membrane.

## 5. Conclusions

Inclusion of INTs into polyamide membranes increased water permeance without negatively affecting salt (sodium) permeability, except for the INT with a hydrophilic pore throat. These findings indicate salt permeability may be manipulated for nanocomposite membranes by tailoring the functionality of the INT pore throat. Water permeance improvements were due to better water diffusion through the confined and straight INT pore throats (primary mechanism). Functionalizing the exterior surfaces of the INTs to be hydrophobic improved their dispersion during the polyamide polymerization step, leading to no visible physical defects in the membrane, as shown by high salt rejection values. Although aligning the INTs vertically, or parallel to the direction of net flow, improved water permeance, it was not substantial.

## Figures and Tables

**Table 1:** Membrane sample designations and preparation notes.

Designation	Description	INT Concentration	INT Modification
TFC	Polyamide thin-film composite	No fillers	No modification
TFN-UA-Me	TFN with unaligned INTs	0.1% *w*/*v* P-Me-INTs	INTs functionalized—Alkyl-Phosphate O.D. and Methyl I.D.
TFN-A-Me	TFN with aligned INTs	0.1% *w*/*v* P-Me-INTs	INTs are fully functionalized—Alkyl-Phosphate O.D. and Methyl I.D.
TFN-A-TEOS	TFN with aligned INTs	0.1% *w*/*v* TEOS-INTs	INTs are grafted with Alkyl-Phosphate across O.D.

**Table 2:** Transmittance peaks at wavelengths corresponding to different groups on the unmodified and modified INTs.

	Si-O Stretching (cm^−1^)	Al-OH-Al (cm^−1^)	Si-OH (cm^−1^)	H_2_O Bending (cm^−1^)	C-H Bending (cm^−1^)	PO_4_^3−^ (cm^−1^)	PO_4_^3−^ (cm^−1^)
INT	1011	3000–3800	2800–3000	1660	n/a	n/a	n/a
TEOS-INT	999	3000–3800	2800–3000	1630	1450	1130	3420
Me-INT	995	3000–3800	2800–3000	1655	1421	n/a	n/a

**Table 3:** Water and sodium permeability characteristics for pure polyamide and nanocomposite membranes.

Sample ID	Internal INT Diameter (nm)	*A* (LMH/bar)	*B* (LMH)	*B*/*A* (bar)	Average Na^+^ Rejection (%)
TFC	No INTs	0.26 ± 0.18	0.02 ± 0.01	3.79	86 ± 5.2
TFN-UA-Me	1.7	1.00 ± 0.16	0.06 ± 0.03	4.61	84 ± 6.5
TFN-A-Me	1.7	1.07 ± 0.29	0.05 ± 0.03	3.91	86 ± 4.5
TFN-A-TEOS	1.0	1.04 ± 0.27	0.14 ± 0.06	9.19	72 ± 9.3

## Data Availability

The raw data supporting the conclusions of this article will be made available by the authors on request.

## Abbreviations

The following abbreviations are used in this manuscript:

A	Aligned
HR-TEM	High-resolution transmission electron microscopy
INT	Imogolite nanotube
Me	Methylated
PSU	Polysulfone
RO	Reserve osmosis
BWRO	Brackish water reverse osmosis
SWRO	Seawater reverse osmosis
SEC	Specific energy consumption
TEOS	Tetraethyl orthosilicate
TFN	Thin-film nanocomposite
UA	Unaligned
